# Nrf2 Is a Protective Factor against Oxidative Stresses Induced by Diesel Exhaust Particle in Allergic Asthma

**DOI:** 10.1155/2013/323607

**Published:** 2013-04-28

**Authors:** Ying-Ji Li, Tomoyuki Kawada, Arata Azuma

**Affiliations:** ^1^Department of Hygiene and Public Health, Graduate School of Medicine, Nippon Medical School, 1-1-5 Sendagi, Bunkyo-ku, Tokyo 113-8602, Japan; ^2^Pulmonary Medicine/Infection and Oncology, Nippon Medical School, 1-1-5 Sendagi, Bunkyo-ku, Tokyo 113-8602, Japan

## Abstract

Epidemiological studies have shown that air pollutants, such as diesel exhaust particle (DEP), are implicated in the increased incidence of allergic airway disorders. *In vitro* studies of molecular mechanisms have focused on the role of reactive oxygen species generated directly and indirectly by the exposure to DEP. Antioxidants effectively reduce the allergic inflammatory effects induced by DEP both *in vitro* and *in vivo*. On the other hand, Nrf2 is a transcription factor essential for the inducible and/or constitutive expression of phase II and antioxidant enzymes. Disruption of *Nrf2* enhances susceptibility to airway inflammatory responses and exacerbation of allergic inflammation induced by DEP in mice. Host responses to DEP are regulated by a balance between antioxidants and proinflammatory responses. Nrf2 may be an important protective factor against oxidative stresses induced by DEP in airway inflammation and allergic asthma and is expected to contribute to chemoprevention against DEP health effects in susceptible individuals.

## 1. Introduction

Air pollution is a complex mixture of compounds in gaseous and particle phases and the strongest evidence from many epidemiological studies linking air pollution with human health effects [1–4] centers around the particulate components. Particles are classified according to their aerodynamic diameter into size fractions such as particulate matter (PM) 10 (thoracic particles, ≤10 *μ*m), PM2.5 (fine particulate matter of diameter, ≤2.5 *μ*m), and ultrafine particles (UFP, fine particulate matter of diameter ≤0.1 *μ*m) [5]. Many epidemiologic studies suggest that PM2.5 is associated with increased respiratory morbidity and mortality [6–8]. Particulate matter includes primary particles that are emitted directly from sources such as fossil-fuel combustion, for example, diesel exhaust particle (DEP), and secondary particles that are generated from gases through chemical reactions [5]. 

Air pollution from motor vehicles has been implicated as an important factor responsible for the increased prevalence of allergic diseases [9]. There is epidemiologic evidence of the impact of diesel exhaust or “near roadway” effects on asthma in humans, particularly children [4]. DEPs are the major components of ambient PM2.5 [10], especially in urban areas, and many *in vivo* and *in vitro* studies have been performed to clarify the association between DEP and pulmonary disorders such as asthma [11–15]. *In vitro *studies have shown that most of the effects of DEP were due to reactive oxygen species (ROS) generated by exposure to DEPs and the subsequent generation of the oxidative stress response within exposed cells [16–21]. 

A nuclear factor, erythroid-derived 2-like 2 (Nfe2l2) or NF-E2-related factor 2 (Nrf2), is a redox-sensitive basic leucine zipper transcription factor that is involved in the transcriptional regulation of many antioxidant genes [22]. Extensive studies have suggested that Nrf2 contributes to protection against various pathologies, including asthma [23–25], chronic obstructive pulmonary disease (COPD) [26], lung fibrosis [27], carcinogenesis [28], atherosclerosis [29], inflammatory disorders [30], and environmental oxidants, including hyperoxia [31, 32] and cigarette smoke [33]. Furthermore, it is implicated that Nrf2 subsidizes host defense through modulation of complex pathways, including well-characterized antioxidant activation in airway inflammation and allergic asthma induced by DEP [21]. A study of a small number of patients with allergic rhinitis showed that susceptibility to the adverse risks induced by DEP is partially dependent on glutathione-S-transferases (GST) genotypes in humans [34]. Oxidant stress seems to be involved in diesel-associated increases in airway inflammation [35] and allergic asthma [36], as evidenced by experiments in *Nrf2* knockout mice. The present review describes recent research demonstrating the effect of Nrf2 in allergic asthma implicated in DEP exposure.

## 2. Characteristics of Diesel Exhaust

DEP produced by diesel engines is a major component of particulate atmospheric pollution, especially in urban areas. DEP has a complex structure characterized by a carbonaceous core with adsorbed organic compounds such as polyaromatic hydrocarbons (PAHs) and quinones. Their small size (0.1–0.3 *μ*m) allows them to penetrate deeply into the respiratory tract and reach the pulmonary alveoli [37]. The PAHs and their oxygenated derivatives (e.g., quinones) have attracted special attention because they are able to participate in the redox cycle and generate ROS in target cells [38]. The main effect observed in healthy human volunteers exposed to DEP is inflammation characterized by an increase of inflammatory cells and chemokines and immunoglobulin E levels in nose lavages [13], which could account for the epidemiological association between chronic exposure to particulate matter and the increase of allergic diseases such as asthma and rhinitis [37]. 

## 3. Nrf2 as a Key Regulator of Phase II Detoxifying Enzyme Genes and Antioxidant-Responsive Genes

Nrf2 was discovered as a ubiquitous transcriptional regulator of antioxidant and detoxification genes. Nrf2 is a transcription factor essential for the inducible and/or constitutive expression of phase II and antioxidant enzymes. For instance, several GST isoforms and NAD(P)H:quinone oxidoreductase 1 (NQO1) were found to be uninducible by xenobiotics in the *Nrf2* germ line mutant mouse [22]. These findings showed that Nrf2 has a major role in transcriptional activation through antioxidant responsive elements.

The expression of phase II detoxifying enzyme genes is clearly induced in wild-type and heterozygous *Nrf2*-knockout mice, but the inducible expression of these genes is markedly reduced in homozygous *Nrf2*-knockout mice [39]. Insufficient induction of cytoprotective enzyme genes causes an increased susceptibility to various xenobiotics [40, 41] and components such as DEP [35, 42]. 

## 4. DEP Induces ROS *In Vitro* and *In Vivo*, and Subsequent Nrf2 Activation Leads to Antioxidant Gene Expression

Studies of molecular mechanisms have focused on the role of ROS generated directly and indirectly by exposure to DEP. ROS play an important role in proinflammatory reactions in airways. Enhanced inflammation involving the activation of alveolar macrophages following DEP exposure leads to the generation of ROS indirectly [43]; however, it is reported that intratracheal exposure to DEP caused the formation of 8-hydroxydeoxyguanosine in the murine lung [44]. DEP chemicals [45] and metals [46] could directly produce ROS such as superoxide and hydroxyl radical. These observations suggest that DEP can generate ROS, leading to oxidative stress-dependent pulmonary damage. DEP induce inflammatory cytokines such as interleukin- (IL-) 8 expression mediated by nuclear factor- (NF-) *κ*B *in vitro*, and these effects are blocked by antioxidant agents such as N-acetyl cysteine (NAC) [16, 18]. These observations also suggest that DEP-induced activation of signal pathways and transcription factors is due to ROS derived primarily from DEP.

*In vitro *research suggests that cytoprotective pathways are induced by the Nrf2 transcription signal pathway at the lowest levels of oxidative stress from DEP and can induce the transcription of antioxidant genes in the earliest level of defense. This may constitute the first tier of a hierarchical oxidative stress response. If these enzymes fail to neutralize the effects of ROS, proinflammatory effects constitute a second tier or superimposed level of oxidative stress. The final tier or superimposed level of oxidative stress is cytotoxicity, including the initiation of programmed cell death [19]. Nrf2 regulates antioxidant defense that is constituted as a main defense action against the proinflammatory and oxidizing effects of DEP [21].

*In vivo* studies with low-level and repeated DEP exposure (100 *μ*g/m^3^) showed that DEP exposure induced airway inflammation in mice [47–50]. Host responses to DEP are regulated by a balance between antioxidant defenses and proinflammatory responses [38]. Studies of two different strains in mice demonstrated that there was a susceptibility difference to DEP exposure, and that certain antioxidant enzymes could be candidates as susceptibility genes [49, 50]. DNA adduct formation has been shown to be accelerated in the lungs of *Nrf2 *knockout mice exposed to DEP [42]. *Nrf2 *knockout mice exposed to low-dose DEP for 8 weeks showed significantly increased airway hyperresponsiveness and counts of lymphocytes and eosinophils, together with increased concentrations of IL-12 and IL-13, and thymus and activation-regulated chemokine (TARC) in bronchoalveolar lavage (BAL) fluid compared with wild-type mice. In contrast, the expression of antioxidant enzyme genes was significantly higher in wild-type mice than in *Nrf2 knockout* mice [35]. This study strongly suggested that DEP-induced oxidative stress and host antioxidant responses were regulated by Nrf2.

It is known that DEP induces and exaggerates allergic airway inflammation *in vivo* [13, 51, 52]. Studies of a murine model of asthma, where mice received repeated low-level exposure to DEP, showed that DEP induced and exaggerated allergic airway inflammation, and NAC treatment reduced these allergic inflammatory responses caused by DEP [53]. *Nrf2 *knockout mice exposed to low-dose DEP showed significantly increased airway hyperresponsiveness and counts of lymphocytes, neutrophils, and eosinophils, together with increased concentrations of TARC in BAL fluid compared to wild-type mice in an asthma model [36]. TARC is a pivotal chemokine for the development of Th2-dominated experimental allergen-induced asthma with eosinophilia and airway hyperresponsiveness [54]. Increased inflammatory cells and PAS staining-positive mucus cell hyperplasia were evident in *Nrf2 *knockout mice. In contrast, the expression of GSH/GSSG (reduced glutathione/oxidized glutathione) was higher in wild-type mice than in *Nrf2 *knockout mice [36]. These results highlighted the role of DEP-induced oxidative stress and host antioxidant responses in the exaggeration of allergic airway inflammation in mice. It has been reported that the responsiveness of the Nrf2-directed antioxidant pathway acts as a major determinant of susceptibility to allergen-mediated asthma [23–25]. These reports suggest that the synergistic effects of oxidative stress caused by DEP and allergens contribute to the major pathway of the exaggeration of allergic asthma.

Disruption of *Nrf2* enhances susceptibility to allergic airway inflammatory responses induced by low-dose DEP (100 *μ*g/m^3^) in mice, but the data did not show any adjuvant activity of DEP for IgE production [36]. The effect of oxidative stresses caused by DEP may be crucial for the induction and exaggeration of allergic airway inflammatory responses due to DEP exposure* in vivo*.

## 5. Future Direction: Nrf2 Is a Key Factor As a Potential Target of Chemoprevention

The production of ROS was closely implicated in airway inflammation, allergy, and asthma; therefore, antioxidants may become a prophylactic strategy against adverse health effects of DEP. Chemoprevention by antioxidants has been reported to reduce the allergic inflammatory effects of DEP in mice [55]. NAC is widely known as an antioxidant drug. DEP-induced oxidants stress and the resultant inflammatory changes were blocked by NAC in asthma model [53]. Sulforaphane, a compound found in broccoli sprouts and broccoli, is also known to be a potent Nrf2 activator and is capable of preventing the toxicity of organic chemicals [56, 57]. Sulforaphane-stimulated phase II enzyme induction inhibits cytokine production by airway epithelial cells stimulated with DEP [58].

Observations from these studies highlight the importance of the Nrf2-antioxidant pathway and may provide new therapeutic strategies for acute respiratory distress syndrome implicated in oxidative stress from DEP exposure.

## 6. Conclusion

Epidemiological, human, and animal experimental studies together suggest that DEP is involved in the recent increased prevalence of allergic diseases. Studies of molecular mechanisms have focused on the role of ROS generated directly and indirectly by exposure to diesel exhaust. Chemoprevention against DEP health effects in susceptible individuals may become a choice for a future environmental protection policy, and Nrf2 is a key potential target of chemoprevention. 
